# Evolution of *EPSPS* double mutation imparting glyphosate resistance in wild poinsettia (*Euphorbia heterophylla* L.)

**DOI:** 10.1371/journal.pone.0238818

**Published:** 2020-09-10

**Authors:** Rafael R. Mendes, Hudson K. Takano, Jéssica F. Leal, Amanda S. Souza, Sarah Morran, Rubem S. Oliveira, Fernando S. Adegas, Todd A. Gaines, Franck E. Dayan

**Affiliations:** 1 Agronomy Department, State University of Maringá, Maringá, PR, Brazil; 2 Department of Agricultural Biology, Colorado State University, Fort Collins, CO, United States of America; 3 Rural Federal University of Rio de Janeiro, Seropédica, RJ, Brazil; 4 Embrapa Soybean, Londrina, PR, Brazil; University of Minnesota, UNITED STATES

## Abstract

The evolution of glyphosate resistance (GR) in weeds is an increasing problem. Glyphosate has been used intensively on wild poinsettia (*Euphorbia heterophylla* L.) populations for at least 20 years in GR crops within South America. We investigated the GR mechanisms in a wild poinsettia population from a soybean field in southern Brazil. The GR population required higher glyphosate doses to achieve 50% control (LD_50_) and 50% dry mass reduction (MR_50_) compared to a glyphosate susceptible (GS) population. The ratio between the LD_50_ and MR_50_ of GR and GS resulted in resistance factors (RF) of 6.9-fold and 6.1-fold, respectively. Shikimate accumulated 6.7 times more in GS than in GR when leaf-discs were incubated with increasing glyphosate concentrations. No differences were found between GR and GS regarding non-target-site mechanisms. Neither population metabolized glyphosate to significant levels following treatment with 850 g ha^-1^ glyphosate. Similar levels of ^14^C-glyphosate uptake and translocation were observed between the two populations. No differences in *EPSPS* expression were found between GS and GR. Two target site mutations were found in all *EPSPS* alleles of homozygous resistant plants: Thr102Ile *+* Pro106Thr (TIPT—mutation). Heterozygous individuals harbored both alleles, wild-type and TIPT. Half of GR individuals were heterozygous, suggesting that resistance is still evolving in the population. A genotyping assay was developed based on the Pro106Thr mutation, demonstrating high efficiency to identify homozygous, heterozygous or wild-type *EPSPS* sequences across different plants. This is the first report of glyphosate-resistant wild-poinsettia harboring an *EPSPS* double mutation (TIPT) in the same plant.

## Introduction

Glyphosate inhibits 5-enolpyruvylshikimate 3-phosphate synthase (EPSPS) in susceptible plants affecting the biosynthesis of aromatic amino acids in the shikimate pathway [1]. This herbicide was first commercialized in 1974 and since the 1990s has been the most used herbicide in the word [2]. The rapid adoption of glyphosate-resistant (GR) crops such as soybean, corn, and cotton contributed to intensify the use of glyphosate worldwide [3]. The possibility to spray a broad-spectrum herbicide on GR crops postemergence simplified weed management and provided an efficient weed control method. Consequently, several weed species have evolved GR in response to the intense selection pressure over more than 50 years of glyphosate commercialization [3]. To date, there are 50 cases of GR in the world, including 31 dicots (*eg*. *Amaranthus palmeri*, *Conyza canadensis*, *Kochia scoparia*) and 19 monocots (*eg*. *Lolium multiflorum*, *Echinochloa colona*, *Digitaria insularis*) that are spread across six different continents [4].

The mechanism by which weeds evolve GR is often associated with altered target site or target site resistance (TSR). Single nucleotide polymorphisms on *EPSPS* sequence resulting in amino acid substitutions at Thr102 or Pro106 usually confer low levels of GR (resistance factor, RF <6-fold) [5]. In contrast, double amino acid substitutions at positions Thr102 and Pro106 in *EPSPS* provide high magnitudes of GR (RF = >8.7-fold) [6]. A goosegrass (*Eleusine indica*) population from Malaysia evolved a double mutation in EPSPS (TIPS, Thr102Ile and Pro106Ser) conferring GR in a weed for the first time [7]. Since then, double mutations these positions have been reported in other weed species such as beggarticks, *Bidens pilosa* (TIPS) [8] and *B*. *subalternans* (TIPT, Thr102Ile and Pro106Thr) [9]. More recently, a GR smooth pigweed (*Amaranthus hybridus*) population evolved a triple amino acid mutation in *EPSPS* (Pro102Ser, Ala103Val and Pro106Ser) [10]. Another important TSR mechanism is associated with the overexpression of *EPSPS* and was first discovered in Palmer amaranth (*Amaranthus palmeri*) [11] and has now been reported in eight species [12]. In this case, GR plants increase the number of *EPSPS* copies and, therefore, EPSPS protein, requiring more glyphosate to inhibit the enzyme and lead to plant death.

Non-target site resistance (NTSR) mechanisms are no less important. Resistant plants can have reduced uptake and/or translocation, which normally decreases the amount of available glyphosate to inhibit EPSPS. For example, GR (RF = 3.7–8.6) in Johnsongrass (*Sorghum halepense*) was associated with lower levels of uptake and translocation compared to susceptible plants [13]. Reduced translocation can also be related to vacuole sequestration, as it is the case in horseweed (*Conyza canadensis*) and ryegrass (*Lolium* spp.) [14, 15]. Glyphosate metabolism is another NTSR mechanism that has been reported in some GR weeds, such as sourgrass (*Digitaria insularis*) [16] and barnyardgrass (*Echinochloa colona*) [17]. The last NTSR mechanism is associated with the “Phoenix” response in which rapid cell death (hypersensitivity) occurs due to the formation of reactive oxygen species (ROS). This mechanism was discovered in giant ragweed (*Ambrosia trifida*) and is considered the most unusual GR mechanism so far [18, 19].

Wild poinsettia (*Euphorbia heterophylla* L.) is a tetraploid species, native of tropical regions and currently one of the most problematic weeds in South America [20, 21]. A recent survey showed that wild poinsettia is present in 20 to 40% of all soybean fields in Brazil, especially in Paraná and Rio Grande do Sul states [22]. Several intrinsic characteristics have allowed wild poinsettia to thrive under no-till systems, such as large seeds, short-day requirement for flowering, and ability to produce up to four generations a year [23]. In soybeans, each day of wild poinsettia-crop coexistence can represent a yield loss of 5.1 kg ha^-1^ [24]. In the 1990’s, wild poinsettia populations evolved resistance to acetolactate synthase (ALS) and protoporphyrinogen oxidase (PPO)-inhibitors when these herbicides were the main tool for postemergence weed control in soybean [25]. While these populations were easily managed by sequential applications of glyphosate once GR crops became available, populations with reduced sensitivity to glyphosate have been observed in a few parts of the country [26, 27]. The objective of this study was to investigate the response of a putative GR wild poinsettia population, and the physiological and molecular mechanisms associated with the resistant phenotype.

## Material and methods

### Plant material, growth and spraying

Seeds from the putative GR wild poinsettia were collected in a soybean field located at Kaloré city (Paraná State, Brazil, 23⁰51’45” S, 51⁰39’58” W). Soybean followed by corn had been planted in this field for 20 years under no-till system. Glyphosate had been applied three times a year in this field (once in corn and twice in soybean). Seeds were collected from 20 plants, bulked, cleaned and stored at 4°C. Following the same method, seeds from a glyphosate susceptible (GS) wild poinsettia population were collected in a field with no history of glyphosate applications located at Engenheiro Coelho (São Paulo State, Brazil, 22⁰05’24” S, 46⁰34’12” W).

Seeds from GR and GS were planted in 200-well flats at 0.5 cm deep. Five to seven d after germination, seedlings were transplanted to 1 L-pots and kept one plant per pot (experimental unit). Flats and pots were filled with potting soil (Growing Mix, Sun Gro, Agawam, MA), kept under greenhouse conditions (25ºC day / 20ºC night, 16 h daylength), and watered daily as needed. Glyphosate (Roundup Transorb R, 480 g ae L^-1^, Monsanto, St. Louis, MO) was sprayed at 480 g ae ha^-1^ on 30 GR plants (12 cm-tall) to eliminate potential GS individuals. Seeds of the surviving plants were collected as described above to obtain the second generation (G2) of GR, which all experiments were performed with. All herbicide treatments were sprayed at 160 L ha^-1^ using a chamber track sprayer (Generation 4, DeVries Manufacturing, Hollandale, LN) equipped with an 8002EVS even flat‐fan nozzle (TeeJet; Spraying Systems Co., Wheaton, IL).

### Whole plant dose-response

The experiment was performed in a factorial design (2 × 11), in which two populations (GS and GR) formed the first factor and glyphosate doses (Roundup PowerMax, 540 g L^-1^, Monsanto) composed the second factor. Glyphosate was sprayed at 0; 65; 130; 270; 540; 720; 1,080; 1,440; 2,160; 4,320; 8,640 g ae ha^-1^ for GS; and 0; 130; 270; 540; 720; 1,080; 1,440; 2,160; 4,320; 8,640; and 17280 g ae ha^-1^ for GR. Plants received the herbicide treatments at the 3-leaf stage (10–12 cm-tall). Visual injury levels were assessed 28 d after treatment (DAT) using 0–100% scale (0%—no symptoms, 100%—plant death). Shoots were collected and dried at 60°C for 72 h before dry mass quantification. The experiment was conducted twice in a completely randomized design with four replications.

### Shikimate accumulation

The shikimate assay was performed as described in the reference [28], with some modifications. Four-mm leaf-discs were excised from the second fully expanded leaves of GS and GR plants and placed in 96-well microtiter plates (one leaf-disc in each well). The wells were filled with 100 μL of 10 mM ammonium phosphate plus 100 μL of glyphosate solution (99% purity, Sigma Aldrich, San Luiz, MO). Glyphosate concentrations were: 0, 16, 31, 63, 125, 250, 500, 1,000 and 2,000 μM. The plates were maintained at 25°C and 500 μmol m^-2^ s^-1^ luminosity in a growth chamber for 16 h. After incubation time, plates were frozen at -20°C for 20 min and transferred to the oven at 60°C for 20 min. The reaction was stopped by adding 25 μL of HCl in each well and heating the plate at 60°C for 50 min. A 25 μL-aliquot from each well was transferred to a new plate containing 100 μL of 0.25% (w v^-1^) periodic acid / 0.25% (w v^-1^) m-periodate. The plates were incubated at 25°C for 90 min, before adding 100 μL of 0.6 N sodium hydroxide/0.22 M sodium sulfite to each well. Absorbance was immediately measured at 380 nm using a spectrophotometer. Background absorbance (rate 0 of each leaf) was subtracted from all wells and a standard shikimate (Sigma Aldrich, Sigma Aldrich, San Luiz, MO) calibration curve was generated for quantification (μg mL^-1^). The experiment was conducted in a complete block design, in which each leaf from GS and GR composed a block. Three leaves from three different plants were used and the experiment was repeated.

### Glyphosate metabolism

Glyphosate and aminomethylphosphonic acid (AMPA) were extracted and analyzed as described elsewhere [9] with some modifications. Plants from GS and GR at the 4–5 leaves stage were sprayed with glyphosate (850 g ae ha^-1^). The whole plants were collected at 24, 48 and 96 h after treatment (HAT). For each timepoint, samples were weighed (0.5 g plant^-1^), washed with distilled water, frozen in liquid nitrogen, ground and stored in 15 mL tubes at -80°C. For glyphosate and AMPA extraction, samples were homogenized in 1 mL distilled water, vortexed for 1 min and shaken for 10 min. The solutions were centrifugated for 10 min (4,000 rpm) and the supernatants were transferred into 2 mL tubes. A new centrifugation was performed at 10,000 rpm for 5 min to precipitate solid residues. Aliquots of 800 μL were used for derivatization with 400 μL of borate solution (5% w v^-1^) and 400 μL of fluorenylmethyloxycarbonyl chloride (10% w v^-1^). The samples were vigorously mixed and incubated at 25°C for 16 h. After the incubation period, samples were centrifuged at 10,000 rpm for 5 min and filtered through 0.2 μm nylon filters (Fisher Scientific, Pittsburg, PA).

Glyphosate and AMPA were measured using liquid chromatography–tandem mass spectrometry (LC–MS/MS; Shimadzu Scientific Instruments, Columbia, MD, USA). The B phase was acetonitrile while the A phase was 10 mM ammonium acetate. A gradient with a flow rate at 0.2 mL min^-1^ was established as follows: 10% B for 2 min; 100% B for 10 min; 10% B for 10.1 min; 10% B for 17 min. A C18 column (100 × 2.1 mm, Alliance Atlantis T3) was used as the stationary phase to separate metabolites according to their solubility. Standard solutions of derivatized AMPA and glyphosate were also injected in each time point for absolute quantification. Two experiments were conducted with three biological replications each.

### ^14^C-Glyphosate uptake and translocation

Seedlings of GS and GR were transplanted into 15 mL Falcon tubes filled with washed sand and fertilizer (Osmocote Classic, Scotts Miracle-Gro, Marysville, OH, 14% N_2_, 14% P_2_O_5_, and 14% K_2_O) equivalent to 0.15 mg cm^-3^ of sand. Plants were kept in a growth chamber (23°C day / 18°C night; 500 μmol m^-2^ s^-1^ light; 10 h daylength). When plants reached the 4-leaf stage, the second last fully expanded leaf was covered with aluminum foil and plants were treated with 540 g ha^-1^ glyphosate plus 0.1% v v^-1^ non-ionic surfactant (NIS). Ten minutes after treatment, the aluminum foil was removed from each covered leaf, before treatment with a solution of 3 μg mL^-1^ glyphosate (equivalent to 540 g ae ha^-1^ in 175 L), 200,000 dpm of ^14^C-glyphosate (phosphonomethyl ^14^C, specific activity: 50–60 mCi mmol ^-1^ American Radiolabeled Chemicals Inc., Saint Louis, MO 63146 USA), and 0.1% NIS. Each leaf received 17 μL of the solution mix (17 droplets of 1 μL), corresponding to 175 L ha^-1^.

Plants were placed in the growth chamber and harvested at 24, 48 and 72 HAT. In each timepoint, the ^14^C-treated leaves were washed with 5 mL of wash solution (10% methanol and 1% NIS) for 30 sec. The roots were washed with 10 mL of distilled water and a 1 mL-aliquot was taken to quantify ^14^C-glyphosate exudation. Wash solutions from roots and leaves were considered as non-absorbed glyphosate. Plant parts were separated (treated leaf, other leaves, and roots) and dried at 50°C for 5 d. Each part was oxidized in a biological oxidizer (OX500; RJ Harvey Instrument Co., Tappan, NY). Samples were measured using a liquid scintillation spectrometry (Packard Tri-carb 2300TR; Packard Instrument, Meriden, CT) after adding 10 mL of scintillation cocktail (Ecoscint XR; National Diagnostics, Atlanta, GA) in each sample.

Absorption values were calculated following Eq 1:

$$\%{}^{14}C\;glyphosate\;absorbed=[{}^{14}C\;ot/\left({}^{14}C\;ot+{}^{14}C\;wl\right)]$$ (1)

Where, ^14^C ot is the amount of total oxidized from plant tissue, ^14^C wl is the amount recuperated from leaf washing. Glyphosate translocation was calculated using Eq 2:

$$\%{}^{14}C\;translocated=100-[{}^{14}C\;pt/\left({}^{14}C\;pt+{}^{14}C\;ot\right)]$$ (2)

Where, ^14^C pt is the total measured in the respective plant tissue, either treated leaf, other leaves, or roots. The average of ^14^C-glyphosate recovered was 90.1%. The experiment was conducted in a complete randomized design with four replications for uptake data and three for translocation data.

### *EPSPS* expression

Young tissue (100 mg) from three GS and ten GR plants was collected and homogenized with TissueLyzer (Qiagen, Germantown, MD). The RNA was extracted with Direct-zol^TM^ RNA MiniPrep Kit, (Zymo Research, Irvine, CA) following the manufacturer’s instructions and quantified with Nanodrop (Thermo Fisher Scientific, Waltham, MA). The cDNA synthesis was performed using 4 μL of iScript RT Supermix (Bio-Rad Laboratories, Hercules,), 1 μL of template RNA (150 ng) and 15 μL of nuclease-free water for 5 min at 25°C, 20 min at 46°C, and 1 min at 95°C.

Primers were designed to amplify short fragments of *EPSPS* (Table 1) and *ALS* (internal standard) from wild poinsettia (reference gene sequences available at Heap, 2020). The short fragments from both genes were amplified, ran in agarose gel (1%) and tested for primer efficiency with a 10-times series of template dilutions [29]. *EPSPS* and *ALS* primers showed 101 and 97% efficiency, respectively. Quantitative PCR (qPCR) was conducted with 1 μL of forward and reverse primers, 1 μL (20 ng) of cDNA, and 12.5 μL of SYBR green Suprmix (Bio Rad Laboratories, Hercules, CA). Three biological and three technical replications from each biotype were used. The qPCR was conducted with the following conditions: 95°C for 10 min, 40 cycles of 95°C for 10 s, and 62°C for 40 s. The subsequent melting curve was performed by increasing the temperature from 59 to 95°C with steps of 0.5°C for 5 s. The relative expression was calculate following the Eq 3 [11]:

$$EPSPS:ALS\;relative\;expression=2^{\left(CtALS-CtEPSPS\right)}$$ (3)

Where, Ct is the cycle threshold.

**Table 1:** Primers used for polymerase chain reaction (PCR) and quantitative PCR (qPCR) of glyphosate susceptible (GS) and glyphosate resistant (GR) wild poinsettia (*Euphorbia heterophylla* L.).

Primer ID	Purpose	Oligo sequence (5’-3’)
EPHH_EPSPS2_F	*EPSPS* sequencing and cloning	CAATCGGATTCTTCTCCTCGCTG
EPHH_EPSPS2_R		TCTCGATTTCTACATCTCCCAGAGC
Express_EPSPS_F	*EPSPS* expression	CTTTATCCGAGGGCACAACT
Express_EPSPS_R		GGGATTACGAGTGGAAGACAAT
Express_ALS_F	*ALS* reference gene	CTAGGATTGTGAGTGAGGCTTT
Express_ALS_R		GAACAGCTAATTGCTGCTGTATATC
KASP_I106_HEX_F	Genotyping assay for Pro106Thr mutation	GAAGGTGACCAAGTTCATGCTGCAGTCAAAG**GA**CGCATTGCT**A**
KASP_P106_FAM_F		GAAGGTCGGAGTCAACGGATTGCAGTCAAAG**TG**CGCATTGCT**G**
KASP_P106I_Uni_R		AACAAGCTATTGTGGAAGGTTGTGG

F: forward, R: reverse. Bolded nucleotides are single nucleotides polymorphism (SNPs) between GS and GR EPSPS sequences used for the Kompetitive Allele Specific PCR genotyping assay (KASP). Underlined nucleotides are HEX or FAM fluorescence markers.

### *EPSPS* cloning and sequencing

Primers were designed to amplify a 519-bp fragment of wild poinsettia *EPSPS* (Table 1) based on the available sequences from the database [4]. The PCR was performed with 12.5 μL of Econotaq Plus 2 × Master Mix (Lucigen, Middleton, WI), 1 μL of forward and reverse primers and 1 μL of the cDNA (20 ng). The reactions were completed to 25 μL with nuclease free water. The PCR conditions were: 97°C for 60 s, 34 cycles of 97°C for 30 s, 62°C for 30 s and 72°C for 90 s, followed by 72°C for 10 min. The PCR products were visualized in agarose gel (1%) and sent for purification and sequencing (Genewiz, South Plainfield, NJ).

The 519-bp fragment was amplified from two GR samples as described above, followed by a 10 min incubation with 0.5 μL of Phusion High Fidelity DNA Taq (New England BioLabs, Ipswish, MA). The PCR product was cloned into Zero Blunt^TM^ TOPO^TM^ plasmid (Invitrogen, Carlsbad, CA), plasmids were heat-shock transformed into competent *E*. *coli* cells, and transformed cells were grown in petri dishes containing Lysogen Broth (LB)-Agar (2.5%) and selected on kanamycin 1 μL g^-1^ for 16 h. One-hundred individual positive colonies were transferred to a new LB solution (2.5% w v^-1^) containing 1 μL g^-1^ kanamycin and grown for additional 16 h. A colony PCR was performed with the same conditions previously described to confirm the fragment insertion and the product was ran in agarose gel (1% w v^-1^). DNA was isolated from 50 positive colonies using a plasmid DNA isolation kit (QiaPrep Spin® miniprep Kit, Qiagen) before sending for sequencing using (sequencing primers specific to the plasmid, specify such as M13F and M13R).

### Genotyping assay for Pro106Thr mutation

A Kompetitive Allele Specific PCR (KASP) genotyping assay was developed to discriminate GS, GR homozygous, and GR heterozygous plants for the Pro106Thr mutation. The assay was based on two allele-specific forward primers differing only by one single nucleotide polymorphism (SNP) to amplify the GR allele (threonine at 106, ACT) or the GS allele (proline at 106, CCT). Primer specifications and the universal (reverse) primer are described in Table 1. Fluorescence labeled oligos were inserted at the 5’ in each forward primer. The specific primers for GS or GR alleles were linked to FAM or HEX fluorescence, respectively (Table 1). The primer mix consisted of 18 μL of FAM primer (100 μM), 18 μL of HEX primer (100 μM), 45 μL of universal reverse primer (100 μM), and 59 μL of nuclease free water. The master mix consisted of 432 μL 2 × KASP (LGC Genomics, Longhton, England) and 32 μL primer mix. Each reaction had 4 μL of master mix and 4 μL of cDNA. The qPCR protocol was: 94°C for 15 min, 10 cycles of 94°C for 20 s, 61°C decreasing to 55°C for 60 s (0.6°C touchdown), followed by 35 cycles of 94°C for 20 s, and 55°C for 60 s. In the end, fluorescence reading was performed with FAM and HEX channels by cooling the plate to 30°C for 10 s. In total, 32 cDNA samples from GR population were analyzed. Two homozygous GS (Thr102 and Pro106) and two homozygous GR (Ile102 and Thr106) samples were included. These control samples were mixed in different proportions of GS:GR (3:1, 1:1, and 1:3) to estimate the number of mutated alleles in the tetraploid genome. Nuclease free water was used as non-template control samples (NTC).

The same 32 genotyped plants at the 3-leaf stage were sprayed with glyphosate at 722 g ha^-1^ (LD_50_ based on the dose response experiments) and plant injury (0–100%) and dry mass were assessed at 35 DAT.

### Data analysis

The dose-response data were subjected to regression analysis and the four-parameter log logistic equation was fitted using the *drc* package in R software [30]. Plant response (visual injury or dry mass reduction) was calculated according to Eq 4:

$$Plant\;response=d+a/\left[1+{\left(x/c\right)}^{b}\right]$$ (4)

Where, *d* is the lowest limit, *a* is the asymptote, *x* is the glyphosate dose (dependent variable), *c* is the *x* value to 50% of a (LD_50_ for plant injury and MR_50_ for dry mass reduction), and *b* is the slope around *c*. The ratio of *c* values between GS and GR was calculated to determine the resistance factor (RF).

Different regression models were fitted for shikimate accumulation in response to glyphosate concentration in GS and GR. The best adjustments were performed with linear function for GS data (*y* = *ax+b*) and logistic function for GR data (*y* = *a* ln(*x*)−*b*). The model selection was based on the goodness of fit and the biological response. For glyphosate metabolism, ^14^C-glyphosate uptake and translocation, and *EPSPS* relative expression experiments, means were compared using t-test (GS vs GR means for each time point). In the genotyping assay, means were expressed in percentage of the highest fluorescence values for FAM and HEX [31]. The means for NTC fluorescence values were subtracted from all samples. One-way ANOVA was used to compare the response of homozygous and heterozygous plants treated with a single glyphosate dose. All statistical analyses were performed using R software (p<0.05).

## Results

### Dose-response experiments

**Fig 1:**
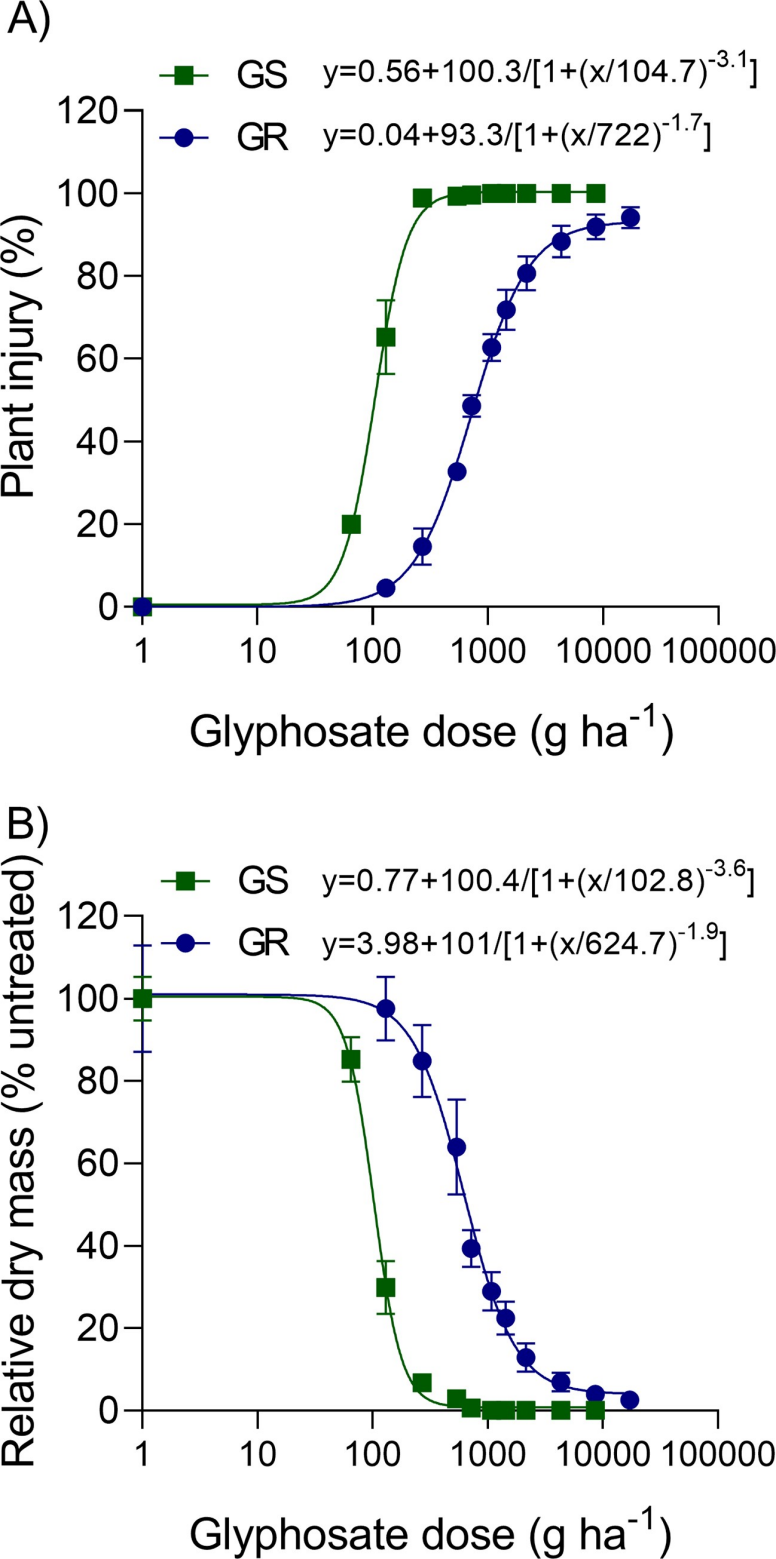
Dose response curves for glyphosate-resistant (GR) and -susceptible (GS) wild poinsettia (*Euphorbia heterophylla*) populations. (A) Plant injury and (B) dry mass. Bars represents standard error of means (n = 8).

Significant differences were observed between GS and GR in the whole-plant dose-response experiments (Fig 1). The LD_50_ was 105 g ha^-1^ for GS and 722 g ha^-1^ for GR resulting in a RF of 6.9-fold (Table 2). Based on MR_50_ values, the RF of GR wild poinsettia was 6.1-fold. Even the highest label rate for wild poinsettia control (2160 g ha^-1^) [32] did not provide high levels of injury (LD_90_) nor efficient mass reduction (MR_90_) on the GR population (Fig 1). In contrast, rates as low as 189 g ha^-1^ (MR_90_) are required to control GS plants. These results confirm that the GR wild poinsettia population is indeed glyphosate resistant.

**Table 2:** Estimated glyphosate doses (g ha^-1^) for 50 and 90% of plant injury (LD) and dry mass reduction (MR), and resistant factors (RF) for wild poinsettia (*Euphorbia heterophylla*) susceptible (GS) and resistant (GR) to glyphosate.

**Population**	**LD**_**50**_	**RF (LD**_**50**_**)**	**MR**_**50**_	**RF (MR**_**50**_**)**
GS	105±7	6.9	103±7	6.1
GR	722±42		625±57	
**Population**	**LD**_**90**_	**RF (LD**_**90**_**)**	**MR**_**90**_	**RF (MR**_**90**_**)**
GS	212±15	12	189±24	10.2
GR	2,543±334		1,924±379	

Model fit: y = a+d/(1+(x/c)^b).

Visual symptoms in GR wild poinsettia after glyphosate treatment were similar to those observed by other researchers [33]. Rates between 500 and 2,000 g ha^-1^ caused significant injury levels even to GR plants until 14 DAT (S1 Fig). After this time period, plants were able to regrow from the axils forming new branches and completing their life cycle. The LD_50_ and MR_50_ values for the GR population (624–721 g ha^-1^) were higher than those observed previously (263–433 g ha^-1^) [33, 34]. While different wild poinsettia populations could have evolved GR in the past [33], no resistance mechanism has been investigated so far.

### Shikimate accumulation

**Fig 2:**
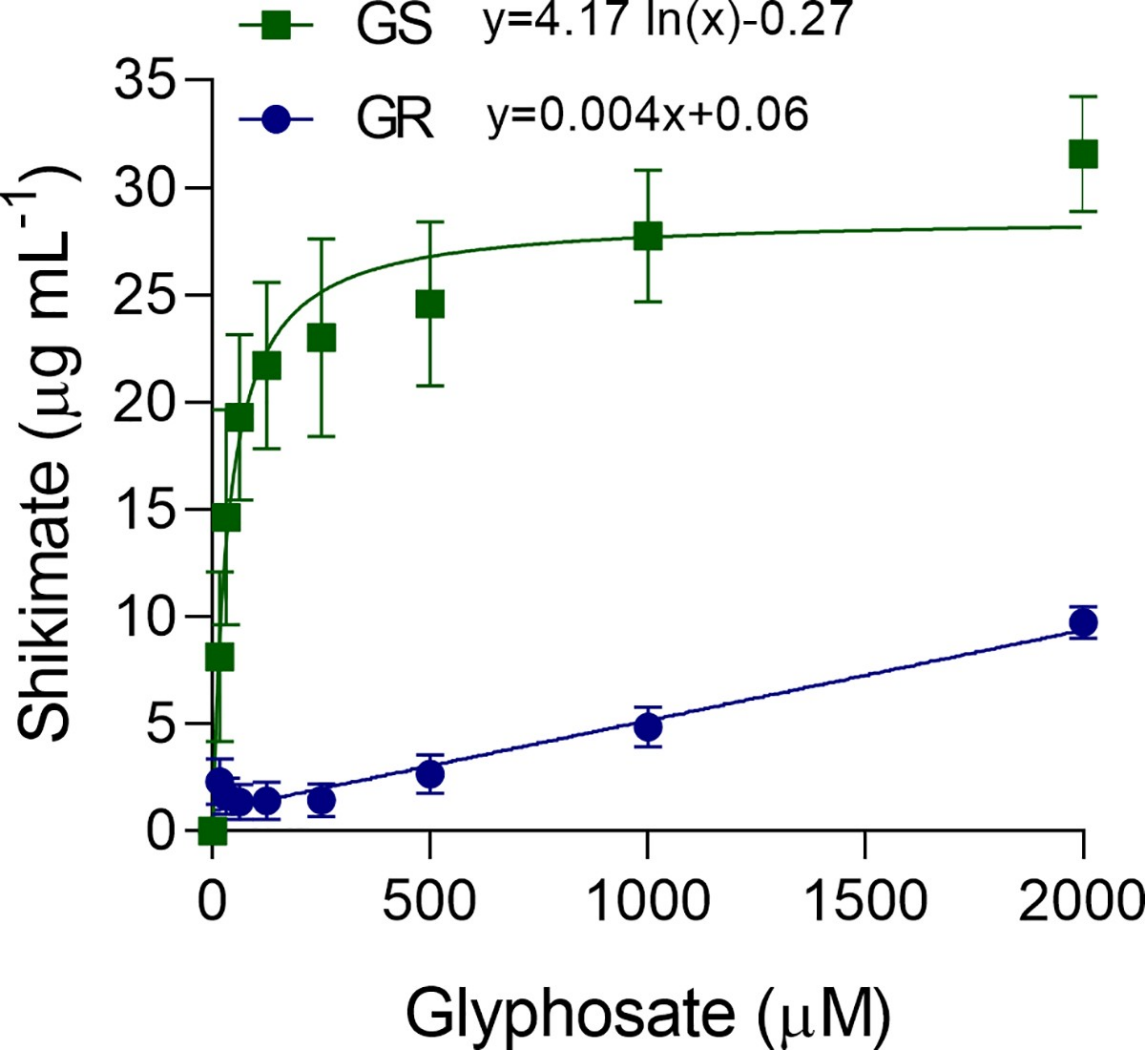
Shikimate accumulation in glyphosate-susceptible (GS) and -resistant (GR) wild poinsettia (*Euphorbia heterophylla*). Data points represent the means ± standard errors (n = 6).

Shikimate accumulated to higher levels in GS compared to GR (Fig 2), consistent with the dose-response experiments. Even with the highest glyphosate concentration (2,000 μM), GR accumulated less than 10 μg mL^-1^ of shikimate, compared to more than 30 μg mL^-1^ in GS (Fig 2). Shikimate accumulation in response to glyphosate concentration followed a logarithmic model for GS (y = 4.17 ln(x)– 0.2669, R^2^ = 0.97) and a linear model for GR (y = 0.004x + 0.0591, R^2^ = 0.94). The average accumulation of shikimate for GS was 21.4 μg mL^-1^, which is 6.7-fold greater than that for GR (3.2 μg mL^-1^), consistent with the RF in the dose-response experiments (6.1 and 6.9-fold).

The shikimate assay is a quick method to discriminate GS and GR weeds [28, 35, 36]. The lower levels of shikimate in GR compared to GS indicates that EPSPS is less affected by glyphosate in GR. This could result from two different hypotheses: 1) A lower amount of glyphosate molecules are reaching EPSPS in GR due to a NTSR mechanism, or 2) EPSPS is either overexpressed or insensitive to glyphosate by a TSR mechanism [37].

### Glyphosate metabolism

No significant differences in glyphosate concentrations were found between GS and GR wild poinsettia across three time points (S2 Fig). Glyphosate concentrations in shoots were approximately 125, 142 and 169 μg g^-1^ for both biotypes at 24, 48 and 72 HAT. No AMPA was measured above the limit of detection (<1 ng g^-1^) for all time points in both GS and GR plants (data not shown). A limited number of species have been documented with the capacity to metabolize glyphosate [3]. A recent study proved that glyphosate can be metabolized by aldo-keto reductase enzymes in a GR barnyardgrass [17]. While glyphosate metabolism can confer GR in some species, neither wild poinsettia population metabolized glyphosate in this experiment.

### ^14^C-Uptake and translocation

**Fig 3:**
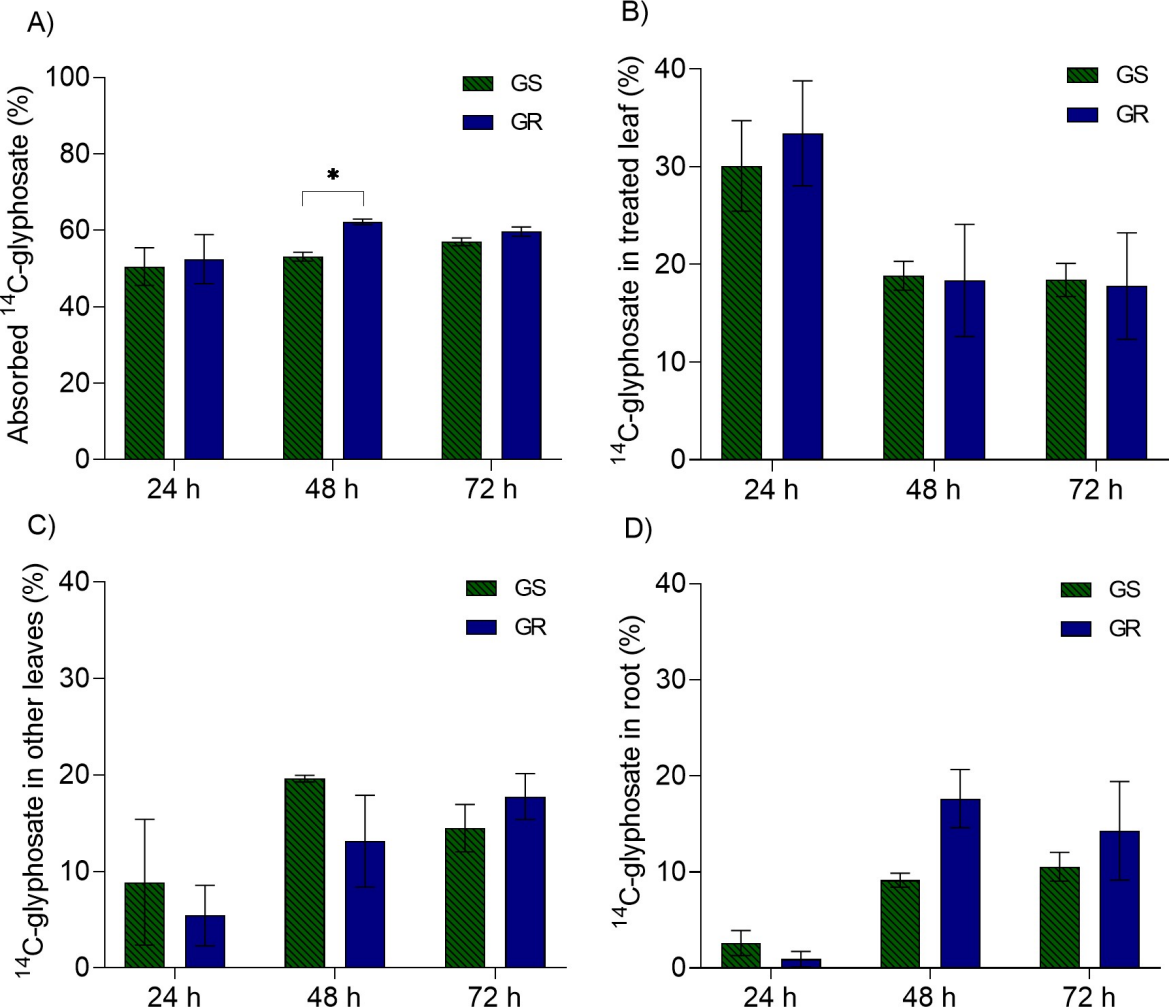
^14^C-Glyphosate uptake and translocation at 24, 48 and 72 h after treatment (HAT) in glyphosate susceptible (GS) and resistant (GR) wild poinsettia (*Euphorbia heterophylla*). (A) absorbed glyphosate, (B) glyphosate in treated leaf, (C) glyphosate in other leaves, and (D) glyphosate in roots. *Means are significantly different by t-test (p<0.05). Bars represent the mean ± standard error (n = 4 for absorption and n = 3 for translocation).

Approximately 55% of ^14^C-glyphosate was absorbed by wild poinsettia plants, with no expressive differences between GS and GR. Only 48 HAT, the uptake was 10% higher in GR than GS plants. However, differences below 15% in just one timepoint are irrelevant to prove some level of differential absorption between populations. Regarding to translocation data, for all timepoints, there were not differences between GS and GR plants, neither for ^14^C-glyphosate present in the treated leaf, nor other leaves and roots (Fig 3).

GR Johnsongrass (*Sorghum halepense*) retained 35–50% more glyphosate in the treated leaf and 15–25% less glyphosate translocated to roots than the susceptible wildtype [13]. Other examples of reduced glyphosate up-take and translocation were also observed in GR waterhemp [38] and ryegrass [39].

### *EPSPS* expression

Similar levels of relative *EPSPS* expression were observed for GS and GR plants, 0.47 and 0.44, respectively (S3 Fig). Pooled means of GS and GR plants were 0.46 (±0.02) and 0.44 (±0.11) of *EPSPS* relative expression, respectively.

*EPSPS* overexpression often results from gene duplication in GR plants, requiring greater amounts of glyphosate to inhibit the pool of EPSPS proteins and lead to plant death [12]. While *EPSPS* gene duplication as a GR mechanism has been reported in eight weed species worldwide [12], it does not contribute to GR in wild poinsettia.

### *EPSPS* sequencing

The *EPSPS* sequences from GS and GR wild poinsettia had 79% similarity to *EPSPS* in *Musa acuminata* (GenBank XM_009420056.2). Four out of 10 GR individuals contained a double amino acid substitution (Thr102Ile and Pro106Thr, TIPT) that is known to affect glyphosate binding to EPSPS. No mutations were found in the remaining six GR plants when analyzed by direct Sanger sequencing of PCR products. Because wild poinsettia is a tetraploid species [21], the 519-bp *EPSPS* fragment encompassing the double mutation region was cloned into *E*. *coli* to identify all possible *EPSPS* alleles.

Two GR individuals were chosen for cloning: One (named individual 1) with only the wild-type allele found in PCR product sequencing (Thr102 + Pro106), and the other (named individual 2) containing Ile102 + Thr106 (based on direct Sanger sequencing of PCR products). In total, 29 clones were sequenced from individual 1 and 17 from individual 2. The individual 1 contained the TIPT allele and a wild-type allele, while only the TIPT allele was found in the individual 2. In the individual 2, 100% of the alleles contained the double TIPT mutation. In contrast, the TIPT double mutation was found in only 34.3% of all alleles analyzed from the individual 1 (Table 3 and S1 File). No cloned sequences showed single mutations at either Thr102 or Pro106.

**Table 3:** Different *EPSPS* alleles from glyphosate-resistant (GR) wild poinsettia individuals.

Individual	Allele	Amino acid position^*a*^												Mutation	Frequency (%)
		99	100	101	102	103	104	105	106	107	108	109	110		
GS^*b*^	wild *EPSPS*				Thr				Pro					-	-
		AAC	GCC	GGG	ACT	GCA	ATG	CGC	CCT	TTG	ACT	GCT	GCA		
Individual 1	*EPSPS*				Ile				Thr					TIPT	66
	*Resistant allele*														
		AAC	GCC	GGG	**ATA**	GCA	ATG	CGC	**ACT**	TTG	ACT	GCT	GCA		
	wild *EPSPS*				Thr				Pro					-	34
		AAC	GCC	GGG	ACT	GCA	ATG	CGC	CCT	TTG	ACT	GCT	GCA		
Individual 2	*EPSPS*				Ile				Thr					TIPT	100
	*Resistant allele*														
		ATC	GCC	GGG	**ATA**	GCA	ATG	CGC	**ACT**	TTG	ACT	GCT	GCA		

^*a*^ Based on *Arabidopsis* sequence.

^*b*^ Sequenced from direct Sanger sequencing.

GS = glyphosate susceptible, TIPT = Thr102Ile + Pro106Thr. Bolded nucleotides are mutant codons.

All cloning sequences are in the S1 File.

The most frequent amino acid substitution resulting in GR occurs at Pro106 on *EPSPS* sequence [6]. At this position, amino acid changes to Ser, Leu, Thr and Ala have been reported [12]. A single mutation at Thr102 has evolved in only one species (*Tridax procumbens*) [5]. To date, only seven from 28 cases of resistance involving TSR mutations in *EPSPS* have been reported with double (Thr102 and Pro106) or triple (Thr102, Ala103 and Pro106) amino acid substitutions [40]. In other species such as beggarticks and goosegrass, double mutations are often present in GR populations but most individuals in the population are heterozygous [7–9].

### Genotyping assay and plant response of homozygous vs heterozygous individuals

**Fig 4:**
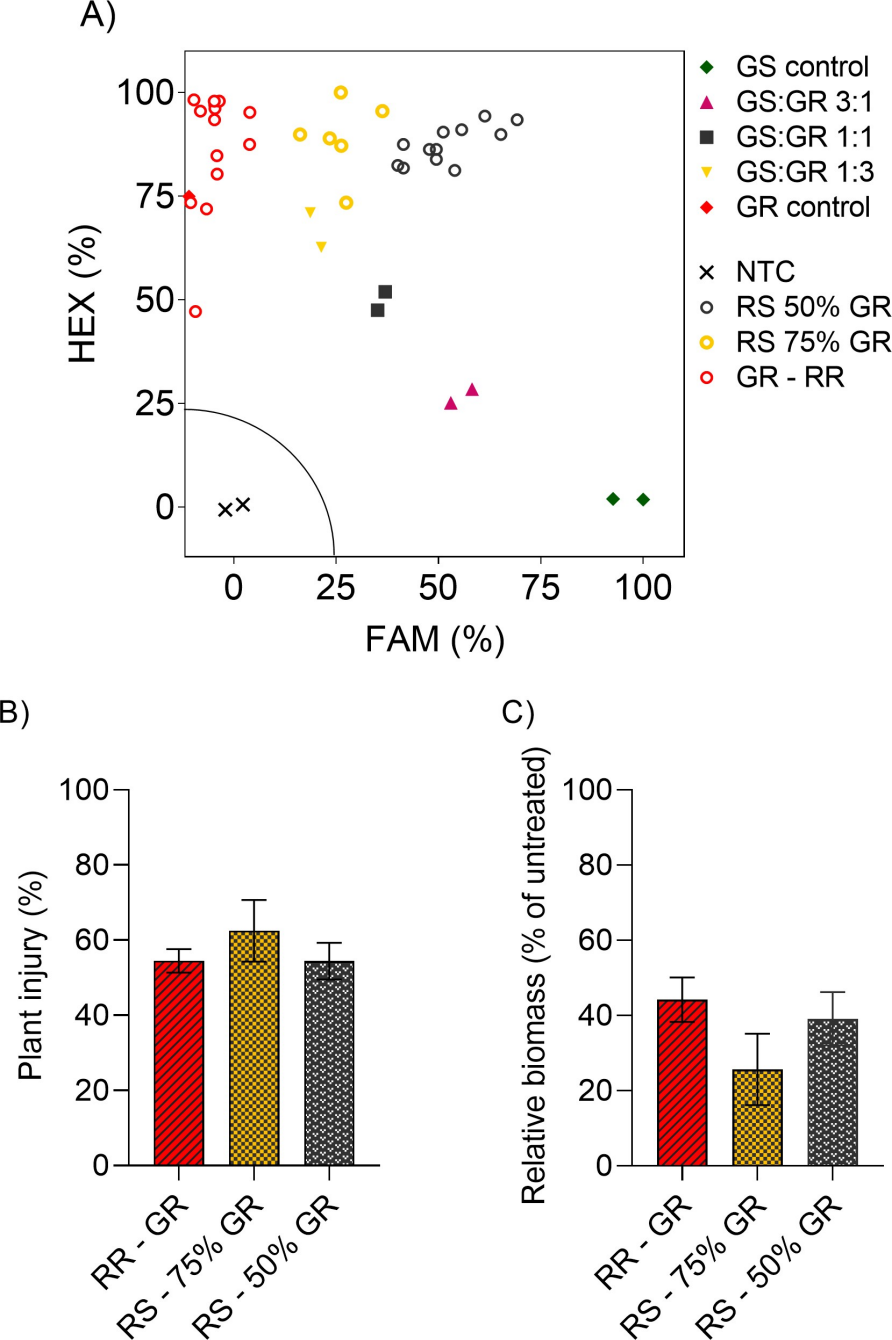
Proportion of homozygous (RR) and heterozygous (RS) individuals within the GR population and their correlation with plant response in glyphosate resistant (GR) wild poinsettia (*Euphorbia heterophylla*). (A) The KASP marker provided three groups of allele frequency based on the *Pro106Thr* mutation: Six heterozygous with 75% of mutant alleles (RS 75%), 12 heterozygous with 50% of mutant alleles (RS 50%), and 14 homozygous with 100% of mutant alleles (RR). (B) Plant injury and (C) Dry mass data in each group of allele frequency (RR, RS 75 and 50% mutant). No differences in glyphosate sensitivity among RR, RS 75% and RS 50% GR individuals (bars represent means ± standard error).

The KASP assay was efficient to discriminate GS, GR homozygous (RR) and GR heterozygous for the TIPT mutation (RS). Based on our sequencing data showing that the same allele carries both Thr102Ile and Pro106Thr (Table 3), we used a molecular marker based only on the Pro106Thr mutation. Out of 32 tested GR plants, 18 (56%) were considered RS and 14 (44%) RR. All RS individuals harbored the TIPT mutation in two (50% of the genome) to three (75% of the genome) out of four alleles (Fig 4A). This assay can be used to monitor many individuals to identify potential resistant plants in other wild poinsettia populations.

## Discussion

Even though we artificially selected the GR population with glyphosate applications for two generations (as described in methods section), the presence of heterozygous plants in the population suggests that the doubles mutation is still segregating in the field. Similarly, 49% of all plants were heterozygous for the TIPS mutation in a goosegrass population from Malaysia [7]. This suggests that homozygous plants could be more resistant than heterozygous individuals. However, RR and RS showed similar response to glyphosate (Fig 4A and 4B).

Target site mutations only at Pro106 do not demonstrate high decrease in glyphosate affinity to EPSPS enzyme (*K*_i_ 1 to 4 μM). In contrast, double mutations at Thr102 and Pro106 always provide *K*_i_ values higher than >38 μM [6]. These numbers are associated with plant response, as observed in goosegrass when Pro106Ser showing 5.6-fold resistance compared to TIPS, expressing >182-fold resistance [7]. While all species found with double mutations have LD_50_ greater than 1,055 g ha^-1^, interestingly, our wild poinsettia has lower LD_50_ (722 g ha^-1^). The *K*_i_ of GR and GS wild poinsettia should be evaluated in the future studies.

A double mutation TIPS in homozygous goosegrass is associated with fitness cost meaning that the individuals carrying this mechanism can slowly disappear in the absence of glyphosate selection pressure [41]. In tetraploid beggarticks species, TIPT or TIPS were found in less than 25% among all EPSPS isoforms [8, 9]. Hence, plants containing heterozygous GR and GS EPSPS can be protected against the selection by fitness cost, an effect known as “gene dosage” in polyploid species [9]. Future analysis will investigate fitness cost comparing RR, RS and GS wild poinsettia.

Wild poinsettia was the second polyploid species harboring TIPT selected in the nature under glyphosate applications. The gene flow between sensitive and resistant plants is a possible in populations of cross-pollinated species [42]. Even the percentage of cross-fertilization has not been determined in wild poinsettia, pollen compatibility was already observed by crossing manually some individuals [43]. Thus, this species may have some level of cross-fertilization which contribute for the pollen-mediated resistance gene flow.

Target site mutations has evolved in wild poinsettia populations: Ser653Leu [44] or Trp574Leu [45] at *ALS* gene, and Arg128Leu at *PPO2* gene [46] causing ALS and PPO resistance, respectively. In this species, target site mechanism imparting GR such as TIPT mutation may increase the risks of multiple resistance evolution, once ALS and PPO resistance populations are widespread across soybean fields in Brazil [26, 27, 33, 47]. Therefore, a diversity of management practices is needed including herbicide mode of action rotation, herbicide mixtures, control of weed dispersion, use of high competitive crops and varieties, and crop rotation [48].

## Conclusions

Wild poinsettia has now evolved GR in Brazil. The resistance mechanisms are not associated with NTSR mechanisms, such as reduced uptake and translocation, nor glyphosate metabolism. *EPSPS* expression levels are similar in GS and GR plants. A double mutation in EPSPS (Thr102Ile + Pro106Thr—TIPT) prevalent in homozygous GR wild poinsettia plants, imparting strong resistance to glyphosate in this population. Heterozygous plants had TIPT in addition to wild-type alleles. Integrated weed management methods must be adopted to mitigate the evolution of multiple herbicide resistant wild poinsettia populations across South America. They should include mode of action rotation, cover crops, minimal weed-crop competition, mechanical control, and weed sanitation practices.

## Supporting information

S1 Fig Response of wild poinsettia (*Euphorbia heterophylla*) plants 14 days after treatment with glyphosate (720 g ae ha^-1^). (PDF)

S2 Fig Glyphosate concentration in glyphosate susceptible (GS) and resistant (GR) wild poinsettia (*Euphorbia heterophylla*) shoots at 24, 48 and 96 h after treatment with 850 g ha^-1^ of glyphosate. Bars represent the mean ± standard error (n = 6). No significant differences were found between GS and GR plants (p<0.05).
(PDF)

S3 Fig *EPSPS* expression (relative to *ALS*) in glyphosate susceptible (GS) and resistant (GR) wild poinsettia (*Euphorbia heterophylla*). Bars represent the mean ± standard error (n = 12). No significant differences between GS and GR were observed (p<0.05).
(PDF)

S1 File Cloning sequences of two glyphosate resistant wild poinsettia individuals. (PDF)

S1 Data (XLSX)
