# Supplementary material for: Evolution of EPSPS double mutation imparting glyphosate resistance in wild poinsettia (Euphorbia heterophylla L.)
Source: PLoS One. 2020 Sep 10;15(9):e0238818. doi: 10.1371/journal.pone.0238818 (PMC7482956; doi:10.1371/journal.pone.0238818)
Supplement: S1 Fig — (PDF) [file pone.0238818.s001.pdf]

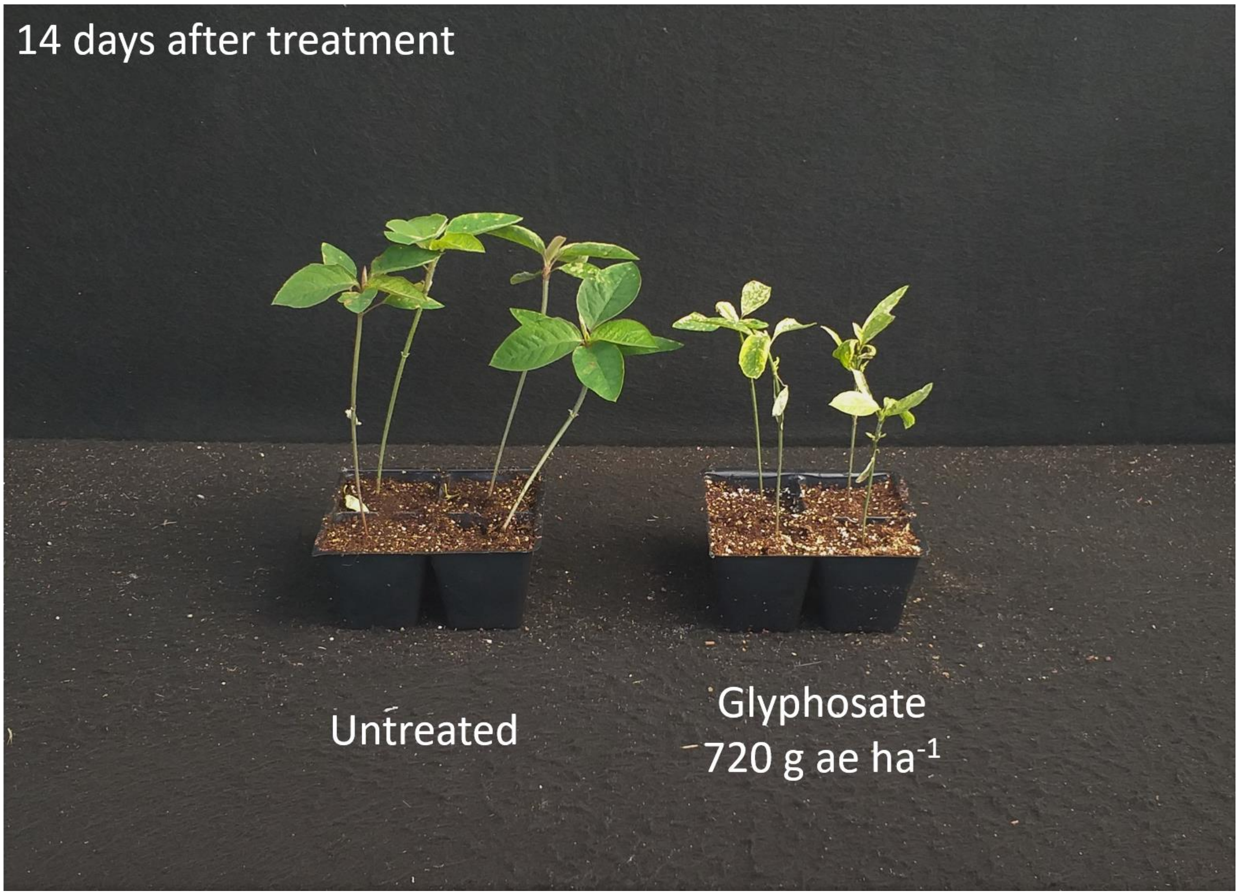

S1 Fig. Response of wild poinsettia (*Euphorbia heterophylla*) plants 14 days after treatment with glyphosate (720 g ae ha^{-1}).
