# Supplementary material for: Evolution of EPSPS double mutation imparting glyphosate resistance in wild poinsettia (Euphorbia heterophylla L.)
Source: PLoS One. 2020 Sep 10;15(9):e0238818. doi: 10.1371/journal.pone.0238818 (PMC7482956; doi:10.1371/journal.pone.0238818)
Supplement: S2 Fig — Bars represent the mean ± standard error (n = 6). No significant differences were found between GS and GR plants (p<0.05). (PDF) [file pone.0238818.s002.pdf]

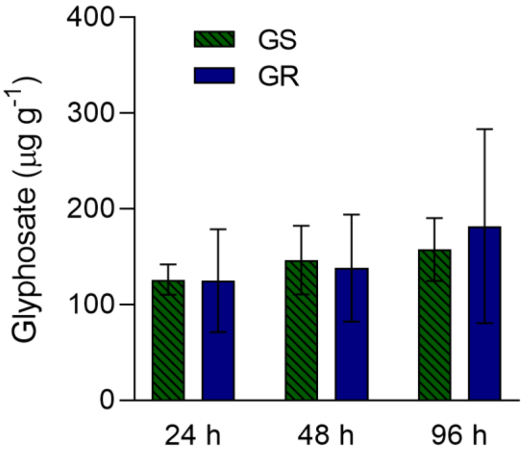

S2 Fig. Glyphosate concentration in glyphosate susceptible (GS) and resistant (GR) wild poinsettia (*Euphorbia heterophylla*) shoots at 24, 48 and 96 h after treatment with 850 g ha^{-1} of glyphosate. Bars represent the mean ± standard error (n = 6). No significant differences were found between GS and GR plants ($p<0.05$).
