# Supplementary material for: Evolution of EPSPS double mutation imparting glyphosate resistance in wild poinsettia (Euphorbia heterophylla L.)
Source: PLoS One. 2020 Sep 10;15(9):e0238818. doi: 10.1371/journal.pone.0238818 (PMC7482956; doi:10.1371/journal.pone.0238818)
Supplement: S3 Fig — Bars represent the mean ± standard error (n = 12). No significant differences between GS and GR were observed (p<0.05). (PDF) [file pone.0238818.s003.pdf]

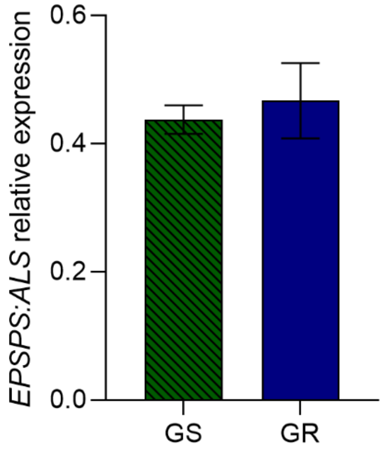

S3 Fig. *EPSPS* expression (relative to *ALS*) in glyphosate susceptible (GS) and resistant (GR) wild poinsettia (*Euphorbia heterophylla*). Bars represent the mean ± standard error (n = 12). No significant differences between GS and GR were observed ($p<0.05$).
