# Supplementary material for: Evolution of EPSPS double mutation imparting glyphosate resistance in wild poinsettia (Euphorbia heterophylla L.)
Source: PLoS One. 2020 Sep 10;15(9):e0238818. doi: 10.1371/journal.pone.0238818 (PMC7482956; doi:10.1371/journal.pone.0238818)
Supplement: S1 File — (PDF) [file pone.0238818.s004.pdf]

S4. Cloning sequences from two glyphosate-resistant wild poinsettia individuals

Individual 1. GR wild poinsettia heterozygous for TIPT mutation (TIPT and wild-type)

```
Clone                  Amino acid position
sample                  102        106
2_  TTTTTCTCGGAATCGCCGGGATAGCAATGCGCACTTTGACTGCTGCAGTTAC
3_  TTTTTCTCGGAAACGCCGGGATAGCAATGCGCACTTTGACTGCTGCAGTTAC
4_  TTTTTCTCGGAAACGCCGGGATAGCAATGCGCACTTTGACTGCTGCAGTTAC
6_  TTTTTCTCGGAAACGCCGGGATAGCAATGCGCACTTTGACTGCTGCAGTTAC
8_  TTTTTCTCGGAAACGCCGGGATAGCAATGCGCACTTTGACTGCTGCAGTTAC
16_ TTTTTCTCGGAAACGCCGGGATAGCAATGCGCACTTTGACTGCTGCAGTTAC
18_ TTTTTCTCGGAAACGCCGGGATAGCAATGCGCACTTTGACTGCTGCAGTTAC
19_ TTTTTCTCGGAAACGCCGGGATAGCAATGCGCACTTTGACTGCTGCAGTTAC
27_ TTTTTCTCGGAAACGCCGGGATAGCAATGCGCACTTTGACTGCTGCAGTTAC
29_ TTTTTCTCGGAAACGCCGGGATAGCAATGCGCACTTTGACTGCTGCAGTTAC
1_  TTTTTCTCGGAAATGCAGGAACTGCAATGCGTCCTTTGACTGCTGCAGTTAC
5_  TTTTTCTCGGAAATGCAGGAACTGCAATGCGTCCTTTGACTGCTGCAGTTAC
7_  TTTTTCTCGGAAATGCAGGAACTGCAATGCGTCCTTTGACTGCTGCAGTTAC
9_  TTTTTCTCGGAAATGCAGGAACTGCAATGCGTCCTTTGACTGCTGCAGTTAC
10_ TTTTTCTCGGAAATGCAGGAACTGCAATGCGTCCTTTGACTGCTGCAGTTAC
11_ TTTTTCTCGGAAATGCAGGAACTGCAATGCGTCCTTTGACTGCTGCAGTTAC
12_ TTTTTCTCGGAAATGCAGGAACTGCAATGCGTCCTTTGACTGCTGCAGTTAC
13_ TTTTTCTCGGAAATGCAGGAACTGCAATGCGTCCTTTGACTGCTGCAGTTAC
14_ TTTTTCTCGGAAATGCAGGAACTGCAATGCGTCCTTTGACTGCTGCAGTTAC
15_ TTTTTCTCGGAAATGCAGGAACTGCAATGCGTCCTTTGACTGCTGCAGTTAC
17_ TTTTTCTCGGAAATGCAGGAACTGCAATGCGTCCTTTGACTGCTGCAGTTAC
20_ TTTTTCTCGGAAATGCAGGAACTGCAATGCGTCCTTTGACTGCTGCAGTTAC
21_ TTTTTCTCGGAAATGCAGGAACTGCAATGCGTCCTTTGACTGCTGCAGTTAC
22_ TTTTTCTCGGAAATGCAGGAACTGCAATGCGTCCTTTGACTGCTGCAGTTAC
23_ TTTTTCTCGGAAATGCAGGAACTGCAATGCGTCCTTTGACTGCTGCAGTTAC
24_ TTTTTCTCGGAAATGCAGGAACTGCAATGCGTCCTTTGACTGCTGCAGTTAC
25_ TTTTTCTCGGAAATGCAGGAACTGCAATGCGTCCTTTGACTGCTGCAGTTAC
26_ TTTTTCTCGGAAATGCAGGAACTGCAATGCGTCCTTTGACTGCTGCAGTTAC
28_ TTTTTCTCGGAAATGCAGGAACTGCAATGCGTCCTTTGACTGCTGCAGTTAC
```

Individual 2. GR wild poinsettia homozygous for TIPT mutation

```
Clone                 Amino acid position
sample                  102        106
1_  TTTTTCTCGGAAACGCCGGGATAGCAATGCGCACTTTGACTGCTGCAGTTAC
3_  TTTTTCTCGGAAACGCCGGGATAGCAATGCGCACTTTGACTGCTGCAGTTAC
6_  TTTTTCTCGGAAACGCCGGGATAGCAATGCGCACTTTGACTGCTGCAGTTAC
7_  TTTTTCTCGGAAACGCCGGGATAGCAATGCGCACTTTGACTGCTGCAGTTAC
8_  TTTTTCTCGGAAACGCCGGGATAGCAATGCGCACTTTGACTGCTGCAGTTAC
9_  TTTTTCTCGGAAACGCCGGGATAGCAATGCGCACTTTGACTGCTGCAGTTAC
10_ TTTTTCTCGGAAACGCCGGGATAGCAATGCGCACTTTGACTGCTGCAGTTAC
11_ TTTTTCTCGGAAACGCCGGGATAGCAATGCGCACTTTGACTGCTGCAGTTAC
13_ TTTTTCTCGGAAACGCCGGGATAGCAATGCGCACTTTGACTGCTGCAGTTAC
14_ TTTTTCTCGGAAACGCCGGGATAGCAATGCGCACTTTGACTGCTGCAGTTAC
15_ TTTTTCTCGGAAACGCCGGGATAGCAATGCGCACTTTGACTGCTGCAGTTAC
16_ TTTATCTCGGAAACGCCGGGATAGCAATGCGCACTTTGACTGCTGCAGTTAC
17_ TTTTTCTCGGAAACGCCGGGATAGCAATGCGCACTTTGACTGCTGCAGTTAC
18_ TTTTTCTCGGAAACGCCGGGATAGCAATGCGCACTTTGACTGCTGCAGTTAC
19_ TTTTTCTCGGAAACGCCGGGATAGCAATGCGCACTTTGACTGCTGCAGTTAC
20_ TTTTTCTCGGAAACGCCGGGATAGCAATGCGCACTTTGACTGCTGCAGTTAC
```
